# Boosting Recombinant Inclusion Body Production—From Classical Fed-Batch Approach to Continuous Cultivation

**DOI:** 10.3389/fbioe.2019.00297

**Published:** 2019-10-31

**Authors:** Julian Kopp, Anna-Maria Kolkmann, Patrick Gwen Veleenturf, Oliver Spadiut, Christoph Herwig, Christoph Slouka

**Affiliations:** ^1^Christian Doppler Laboratory for Mechanistic and Physiological Methods for Improved Bioprocesses, Institute of Chemical, Environmental and Bioscience Engineering, Vienna University of Technology, Vienna, Austria; ^2^Research Division Biochemical Engineering, Institute of Chemical, Environmental and Bioscience Engineering, Vienna University of Technology, Vienna, Austria

**Keywords:** *Escherichia coli*, fed-batch, continuous biomanufacturing, stirred-tank reactor cascade, inclusion bodies

## Abstract

State of the art microbial recombinant protein production is regularly performed in fed-batch based cultivations. However, these cultivations suffer from highly time-dependent changes in productivity and product quality, leading to high variations in the downstream process. Continuous biomanufacturing offers the possibility of a time independent process, boosting the time-space-yield of the recombinantly produced protein and further reducing costs for production, also as downstream gets more predictive. In the current work, the continuous production of a pharmaceutically relevant protein in form of an inclusion body in *E. coli* BL21(DE3) was investigated in single vessel cultivations by varying dilution rates using glycerol as carbon source, inducer (lactose or IPTG) and respective inducer concentrations. Attempts to increase low specific productivities observed in single vessel continuous cultivations, led to the establishment of a continuously operated cascade of two stirred tank reactors to spatially separate biomass formation from recombinant protein production. Process performance was substantially improved compared to a single vessel chemostat culture, as specific productivity and space-time yield were boosted using an optimized cascaded process by about a factor of 100. This study shows the potential of a two-stage continuous process as promising alternative to benchmark fed-batch processes achieving constant inclusion body production at a time-independent level.

## Introduction

About one third of all recombinant pharmaceuticals in industry is expressed in the Gram-negative bacterium *E. coli* (Walsh, 2010; Gupta and Shukla, 2016). Usage of *E. coli* as host can be advantageous over other organisms due to its well-known genetics, high growth rates as well as relatively inexpensive culture media and process operation (Huang et al., 2012). Recombinant proteins in *E. coli* are often produced in insoluble aggregated form, known as so called inclusion bodies (IBs). Even though IBs have originally been believed to be waste products produced by bacteria (Garcia-Fruitos et al., 2012) the formation of IBs provides unique opportunities in cultivation strategies as IBs show higher densities compared to cellular impurities (Schein, 1989). Reports show that purity up to 95% of the target proteins were found in IBs (Georgiou and Valax, 1999). Even though long and cost intensive downstream applications cause the bottle neck in IB-based processes the overall time-space-yield tends to be favorable for IB-production when being compared to periplasmic or soluble protein production (Jagschies et al., 2018). *E. coli* BL21(DE3), is often used in industry due to low acetate formation and high production rates as an effect of the integrated T7-polymerase (Steen et al., 1986; Studier and Moffatt, 1986; Studier et al., 1990; Dubendorff and Studier, 1991; Neubauer and Hofmann, 1994; Lyakhov et al., 1998), which is transcribed from the lac-operon and allows expression from pET plasmids using the T7-promoter (Dubendorff and Studier, 1991; Marbach and Bettenbrock, 2011; Wurm et al., 2016). Here induction can be performed either with lactose (converted to allolactose) or the well-known structural analog isopropyl β-d-1 thiogalactopyranoside (IPTG) (Neubauer and Hofmann, 1994; Wurm et al., 2016). However, induction with IPTG stresses the host cells, and IPTG in higher concentrations (higher than 1 mmol/L) is even referred of being toxic at elevated induction times (Neubauer and Hofmann, 1994; Donovan et al., 1996; Viitanen et al., 2003; Marbach and Bettenbrock, 2011). Using either IPTG (Marisch et al., 2013) or lactose (Neubauer et al., 1992; Neubauer and Hofmann, 1994; Ukkonen et al., 2013) as inducer resulted in higher product yields when compared to other inducer supplies (Marschall et al., 2016). “Soft induction” performed with lactose showed especially promising results (Neubauer et al., 1992; Neubauer and Hofmann, 1994; Blommel et al., 2007; Kopp et al., 2017). As lactose can be partly metabolized in *E. coli* BL21(DE3), it does not stress the cells as much as IPTG (Dvorak et al., 2015). For the production of soluble proteins and products located in the periplasm, induction with lactose is referred to be a sufficient method (Wurm et al., 2016), but it has also been shown that lactose provided promising results for IB-production (Wurm et al., 2016, 2017a). As fed-batch cultivations suffer from highly different metabolic loads, which often occur when IPTG is used as inducer (Neubauer et al., 2003), the constant feeding of a soft inducer could help to eliminate time-dependent variations and achieve stable productivity instead.

In general, glucose is used as main carbon source for growth of *E. coli*. However, as glucose in pure form is quite expensive, industry aims for cheap complex media or inexpensive carbon sources for defined media. Glycerol, first noticed in biotechnology as a waste product in the biodiesel production (Martínez-Gómez et al., 2012), has shown beneficial effects in *E. coli* cultivations regarding biomass-substrate yields (Blommel et al., 2007; Ukkonen et al., 2013) and specific productivities (Kopp et al., 2017). As excess glycerol generated cannot be disposed of in the environment, the application of glycerol as a carbon and energy source for microbial growth in industrial microbiology may help to solve environmental problems (da Silva et al., 2009). Unlike glucose, glycerol is an “energy-poor” carbon source (Deutscher et al., 2006) and should not imply catabolite repression when organism are cultivated concomitantly with lactose (Lin, 1976). However, in a previous study we found out, that the inducer uptake rate is rather a function of product formation and phenomena of existing or non-existing catabolite repression are only seen when no product formation occurred (Kopp et al., 2017).

Batch/Fed-batch bioprocessing is today's state of the art in industrial microbial production of biopharmaceuticals (Croughan et al., 2015). The application of chemostat reactors was first described by Monod (1950); Novick and Szilard (1950); Rinas and Bailey (1992). However, establishing continuous biomanufacturing seems to be getting more interesting again, because of optimum use of installed assets and highest achievable time space yields (Hoskisson and Hobbs, 2005). Chemostat systems provide conditions, where cells are maintained in a steady-state growth environment (dx/dt = 0) (Hoskisson and Hobbs, 2005). Supplying the cells with a constant flow of nutrients combined with simultaneous removal of spent culture medium at a defined rate enables carbon-limited definition of cell growth (Rinas and Bailey, 1992). Establishing long steady-state conditions is believed to provide stable volumetric productivities and high space-time yields (STY), achieved by lower set-up and running costs such as a straightforward process scalability due to a practicably reduced equipment size compared to fed-batch processes (Walther et al., 2015; Peebo and Neubauer, 2018). Smaller production volumes can support process development, due to rather uncomplicated upscale processing (Croughan et al., 2015) and less complicated plant manufacturing. As a consequence this reduced risk of technology transfer could ease clinical production and commercial manufacturing (Daszkowski, 2013). Although various highly efficient microbial continuous processes for the production of recombinant proteins are described in literature (Domingues et al., 2005; Paulova et al., 2012; Schmideder and Weuster-Botz, 2017), the only known example of a continuous industrial recombinant process dates back to the 1990s, when insulin was produced using the host *S. cerevisiae* (Diers et al., 1991), however process stability was problematic. In particular, continuous recombinant expression of intracellular proteins is challenging because a separation of the retention times of the product and the biocatalyst is impossible, causing a strong metabolic/product burden if strong promotors are applied (Studier and Moffatt, 1986; Striedner et al., 2003; Tripathi, 2009). In batch cultivations, the separation of cell growth and recombinant protein production is done on a time-dependent manner, as biomass growth can be performed in batch and fed-batch mode without additional production stress and induced once the desired biomass concentration is achieved. As continuous cultivation is time-independent, the growth of cells is inhibited and genetic instabilities, such as plasmid loss, occur due to strong selection pressure (Rhee and Schügerl, 1998; Croughan et al., 2015). Moreover, continuous bioprocesses suffer from comparatively low product concentrations (Schmideder and Weuster-Botz, 2017). Nowadays, novel selection marker strategies (Selvamani et al., 2014), tunable protein expression (Marschall et al., 2016) and bioprocess strategies (Gupta et al., 2001) have been developed to face the challenges being associated with long-term cultivations such as stable systems, sterility and genetic instabilities. One possibility to reduce the metabolic burden in continuous cultivations is the decoupling of growth and recombinant protein production by applying a cascade of two sequentially operated continuous bioreactors (Schmideder and Weuster-Botz, 2017). During biomass formation in the first reactor, cells should be growing burden-free. The non-induced biomass from the first reactor is transferred to a second reactor where the recombinant protein production is induced at much higher cell densities, when compared to a one-compartment system. Using this system, a highly concentrated feed can be used for the induction in the second reactor, giving the chance of applying similar feeding systems used in fed-batch systems. Additionally, each reactor can be operated at optimal conditions (such as pH and temperature) for growth and protein production plus the whole system can be varied in residence times. However, cascaded continuous cultivation is restricted to academic studies so far (Brown et al., 1985; Park et al., 1989, 1990; Park and Ryu, 1990; Hortacsu and Ryu, 1991; Kim and Ryu, 1991; Akhnoukh et al., 1996; Baheri et al., 1996).

In this study, we present the development of an industrial based continuous inclusion body process to produce a pharmaceutically relevant product in *E. coli* BL21(DE3). The glycerol-lactose system being established for fed-batch cultivations is applied in continuous cultivation for the first first-time, providing a suitable alternative to optimized fed-batch processes. By varying cultivation strategies in the cascaded system, recombinant protein formation could be efficiently boosted.

We also want to highlight that the cascaded system, using the cultivation strategies above, is superior to previous cascaded cultivations in terms of volumetric productivity **(**Schmideder and Weuster-Botz, 2017**)** and could be a promising alternative to common batch systems as reduced plant dimensions in combination with the usage of relatively cheap carbon sources could significantly reduce the costs for industrial applications.

## Materials and Methods

### Bioreactor Cultivations

All cultivations were carried out with the strain *E. coli* BL21(DE3) consisting of a pet30a plasmid system. The target protein was linked to a N-pro fusion tag (Achmüller et al., 2007). As the given protein is currently under patenting procedure at the industrial partner no further information can be given on the used protein.

All bioreactor and preculture cultivations were carried out using a defined minimal medium referred to DeLisa et al. (1999). Batch media and the preculture media contained of the same ingredients with different amounts of glycerol, respectively, 20 and 8 g/L.

As the used pET30a-system consisted of a Kanamycin resistance gene, antibiotic was added throughout all fermentations, trying to prevent plasmid loss, resulting in a final concentration of 0.02 g L^−1^. All precultures were performed using 500 mL high yield flasks. They were inoculated with 1.5 mL of bacteria solution stored in cryos at −80°C and subsequently cultivated for 20 h at 230 rpm in an Infors HR Multitron shaker (Infors, Bottmingen Switzerland) at 37°C.

All cultivations were either performed in a DASBox Mini bioreactor 4-parallel fermenter system (max. working volume [=wV].: 250 mL; Eppendorf, Hamburg, Germany) or in a Labfors 4 bioreactor (max. wV: 1 or 5 L; Infors HT, Bottmingen, Switzerland). For the two-stage cultivations, two continuously operated stirred-tank reactors were connected by a transfer tube coupled to a pump (Ismatec, Wertheim, Germany) (**Figure 2**). For measuring the CO_2_ and O_2_ flows, either a DASGIP-GA gas analyzer (Eppendorf, Hamburg, Germany) or BlueSens Gas sensors (BlueSens Gas analytics, Herten, Germany) were used.

Distributed process control was established using either the PIMS Lucullus (Securecell, Swiss) or DASware-control, respectively. pH was kept constant at 6.7 throughout all cultivations and controlled using a base only control (12.5% NH_4_OH), while acid (5% H_3_PO_4_) was added manually, when necessary. The pH was monitored using an EasyFerm Plus pH-sensor (Hamilton, Reno, NV, USA). The reactors were continuously stirred at 1,000 or 1,400 rpm, respectively. Aeration was absolved using a mixture of pressurized air and pure oxygen at 1 to 3 vvm. As the cultivation volume of the reactor was adjusted via an immersion tube connected to a bleed-pump, no stirrer cascade was implied as volumes and residence times would alter at higher rotation levels. Addition of pure oxygen purged the need for a stirrer cascade, as the ratios of the airflow were mixed together so that the dissolved oxygen (dO_2_) was always higher than 30%. The dissolved oxygen was monitored using a fluorescence dissolved oxygen electrode Visiferm DO (Hamilton, Reno, NV, USA).

### Cultivation Scheme

**Figure 1:**
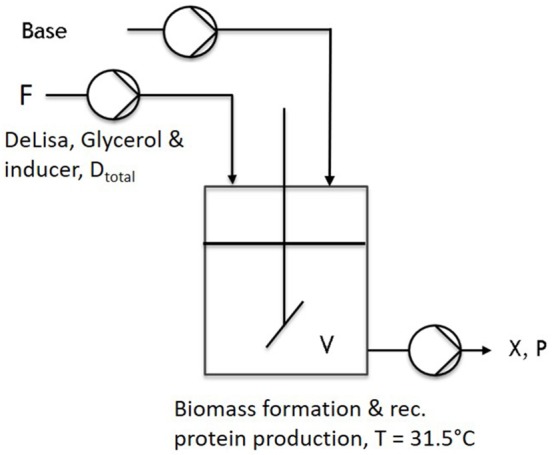
Schematic setup of a common chemostat used throughout first cultivations.

**Figure 2:**
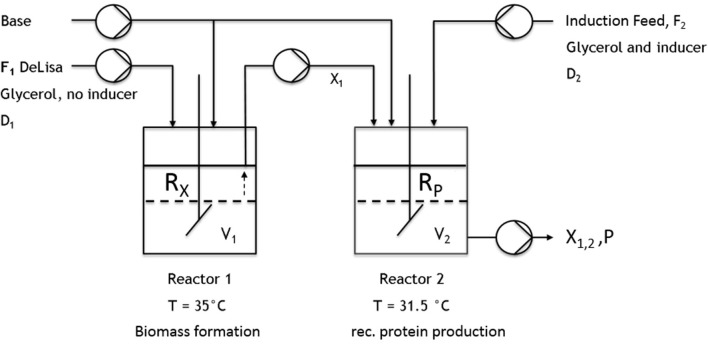
Schematic setup of the implemented cascade using two continuously operated stirred-tank reactors. The first reactor was used for biomass formation (no inducer). The culture broth was then transferred to the second reactor, where recombinant protein production was induced.

During one-stage continuous cultivations (Figure 1), a batch phase was applied to gain biomass in a first stage, keeping (20 g L^−1^ glycerol) the temperature constant at 37°C. The chemostat system was applied with the start of the induction phase. Therefore, the feed was changed for continuous application, ensuring continuous supply of a mixed-feed (with specific glycerol uptake rate of q_s,gly_ = 0.2 g/g/h and a specific inducer uptake rate of q_s,lac_ = 0.1 g/g/h), as well as the start of the bleed pump. Volume was adjusted via an immersion tube adjusted to right height to the liquid surface, ensuring that the bleed pump was working at higher rate than the feed pump. As the bleed pump ensured a complete deduction of the fed media, D was calculated as a flow in, monitored via multiple scales, and the process system Lucullus (Securecell, Schlieren, Switzerland). During cascaded continuous cultivation (250 mL wV in DAS-Box system)—sketch given in Figure 2—a batch phase for biomass formation was performed in both reactors. This was followed by a non-induced continuous phase, lasting over night for approximately 2 residence times (depending on the dilution rate) trying to achieve a steady state. Once the equilibrium state was reached, visible by monitoring the off-gas values, a feed containing glycerol (50 g/l) for the first feed and the respective glycerol/inducer feed (500 g/L glycerol 93 g/L lactose monohydrate) was continuously supplied to the second reactor. For biomass formation during continuous cultivation the temperature was set to 35°C. For protein production the temperature of the second vessel, was lowered to 31.5°C as optimized results were obtained at this temperature (Slouka et al., 2018). The inducer concentration in the mixed feed was calculated in order to ensure a maximum specific uptake rate of lactose of q_s,lac_ = 0.23 g g^−1^ h^−1^ (Kopp et al., 2017).

### Process Analytics

Samples were always taken upon the end of the batch-phase and after the non-induced fed-batch and regularly during continuous process mode. In the induction period, samples were taken every 3 h during the first day of induction, to monitor the first protein production stages, followed by daily sampling, which was increased to two times per day if necessary, for the whole duration of the cultivation. Biomass was measured using optical density (OD_600_) and gravimetrically by weighing the dry cell weight (DCW), while flow cytometry analysis (FCM) was used for the determination of cell-death. OD_600_ was measured in duplicates using a Genesys 20 photometer (Thermo Scientific, Waltham, MA, USA). Since the linear range of the used photometer was between 0.2 and 0.8 [AU], samples were diluted with dH_2_O to stay within the given range. The DCW was determined by centrifuging 1 mL of homogenous sample solution in a pre-tared 2 mL Eppendorf-Safe-Lock Tube (Eppendorf, Hamburg, Germany). Centrifugation conditions were at 10,000 rpm for 10 min at 4°C. After centrifugation, the supernatant was split into two different fractions. One fraction was immediately used for automated at-line measurement of glycerol, phosphate nitrogen and acetate using a Cubian XC (Optocell, Bielefeld, Germany). The second fraction was frozen at −20°C and later used to determine, e.g., sugar accumulation. For washing purpose, the pellet was re-suspended with 1 mL of 0.9% NaCl solution and centrifuged at the same conditions. Afterwards, the pellet was dried for at least 48 h at 105°C and evaluated gravimetrically. DCW measurements were performed in at least three replicates. Samples for FCM were diluted 1:100 with 0.9% NaCl solution, stored at 4°C and measured every day. The measurement was performed using the software Cube 8 (Sysmex, Partec, Grlitz, Germany) according to Langemann et al. (2016) using DiBAC4(3) (bis-(1,3-dibutylbarbituricacid-trimethineoxonol) and Rh414 dye. Rh414 binds to the plasma membrane and visualizes all cells by reducing the background signal, while DiBAC4(3) is sensitive to the plasma membrane potential and thus enables the distinction between viable and non-viable cells.

Sugar concentrations of feed and fermentation broth were measured via HPLC (Thermo Scientific, Waltham, MA, USA) using a Supelcogel-column (Eluent: 0.1% H_3_PO_4_; Flow: 0.5 mL/min). Glycerol accumulation, lactose decrease, and galactose accumulation were monitored using this method. Standards had a concentration of 1, 5, 10, 20, 30, 40, and 50 g L^−1^ of the respective used carbon sources throughout fermentation. The HPLC run lasted always for 25 min and chromatograms were analyzed using a Chromeleon Software (Thermo Scientific, Waltham, MA, USA).

### Product Analytics

Product samples were taken after the start of the induction phase. Therefore, 5 mL broth was pipetted in a 50 mL falcon tube and centrifuged for 10 min at 4,800 rpm at 4°C. The supernatant was discarded while the pellet was frozen at −20°C. Afterwards the samples were disrupted for homogenization as follows: The pellets were re-suspended in a lysis buffer (0.1 M TRIS, 10 mM EDTA, pH = 7.4) according to the dry cell weight. After suspending the cells with a disperser (T 10 basic ULTRA-TURRAX®, Staufen, Germany) they were treated with an EmusiflexC3 Homogenizer (Avestin, Ottowa, ON, USA) at 1,300 bar. Homogenization always lasted for at least ten passages, ensuring complete cell disruption. After washing the pellets twice with ultrapure water, they were aliquoted and then centrifuged (14,000 rpm, 10 min 4°C). The supernatant was discarded, and the pellets were stored at −20°C until further processing.

For titer measurements of the N-pro-fused protein, the pellets were dissolved using a solubilization buffer (7.5 M Guanidine Hydrochloride, 62 mM Tris at pH = 8). The filtered samples were quantified via RP-HPLC (Thermo Scientific, Waltham, MA, USA) using a Nucleosil-column. The eluent was a gradient mixture of water with 0.1% TFA (tri-fluoric-acid) and acetonitrile mixed with 0.1% TFA with a flow of 3 mL min^−1^. Standard concentrations were 50, 140, 225, 320, 500, and 1,000 mg mL^−1^ of an industrial supplied reference standard.

## Results

### From Fed-Batch to Continuous Cultivation

**Figure 3:**
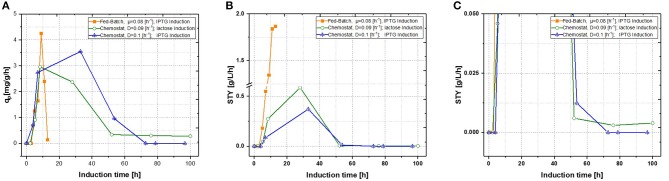
Comparison of two single-stage chemostat systems induced with lactose or IPTG with a state-of-the-art fed-batch **(A)** specific productivity as function of induction time; **(B)** space-time yield as function of induction time. **(C)** Zoom of the space time yield comparing the effects of different inducers within the chemostat cultivations.

Production of recombinant proteins in *E. coli* is usually carried out as a fed-batch process, which generally consists of a biomass production phase followed by an induction phase for producing the desired recombinant product. Since this approach leads to highly time dependent changes in critical quality attributes (CQAs), such as product titer, purity and size (Garcia-Fruitos et al., 2012; Slouka et al., 2018), the aim of this study was to create a process, providing time-independent product formation. Critical process parameters (CPP), like optimal temperature for growth and recombinant protein expression as well as carbon source and inducer concentrations, were identified in recent fed-batch studies (Kopp et al., 2017). This knowledge paved the way for the first continuous cultivations. First chemostats were operated at dilution rates of approximately D = 0.1 [h^−1^], as recombinant protein production showed highest specific inducer uptake rates (=_qs, lac_) in previously established fed batch cultivations (Wurm et al., 2016). As shown in Figure 3, recombinant protein production reached higher specific productivity using IPTG as an inducer, though after 55 h of induction, product formation dropped down completely. For fed-batch cultivations, q_p_ and space-time yield do increase within short time spans, however productivity declines within the timespan of induction phase. This effect can be explained by cell lysis and reduced capacity being caused by the high metabolic burden exerted onto host cells (Ceroni et al., 2015; Slouka et al., 2019).

Calculations were performed establishing rates between the corresponding sampling intervals. q_p_ was calculated using titer measurements from HPLC and the active measured biomass or viable cell concentration (= VCC) in between the sampling interval given in Equations (1–3):

(1) $$Qp\;=\;\frac{Titer\;HPLC\;\left[\frac{g}{L}\right]{}^{*}Reactor\;Volume\;[L]}{\frac{Average\left(DCW_{1,2}\right)\;[g]}{Sampling\;time\;2\;\left[h\right]\;-\;sampling\;time\;1\;[h]}}$$

(2) $$\begin{array}{l} DCW_{1}\;=\;\left[g\right]=(DCW\left[\frac{g}{L}\right]\\ \;-\left(\%dead\;cells^{*}DCW\left[\frac{g}{L}\right]\right)){}^{*}Reactor\;Volume[L] \end{array}$$

(3) $$\begin{array}{l} STY\;=\;\left[\frac{\frac{g}{L}}{h}\right]\;=\;Titer\;HPLC\;\left[\frac{g}{L}\right]/t\;\left[h\right] \end{array}$$

With q_p_ being the specific productivity in g/g/h and STY the time space yield (in g/L/h), which was calculated with product titer measurements derived from HPLC and then calculated for the corresponding time phase. IPTG induction reaches a short time induction maximum after 40 h, hence production stops completely at ongoing induction times. This would correlate to the well-known knowledge, that IPTG cannot be metabolized, eventually creating toxic effects onto *E. coli*, therefore decreasing recombinant product formation at evaluated cultivation times (Dvorak et al., 2015). Performing induction with lactose nearly reached same maximum productivity as performed with IPTG induction, however product formation never dropped down to zero, when comparing to the zoom in Figure 3C. Additionally, no lactose accumulation was measured throughout the induction phase of the chemostat (not shown), leaving room for optimization by adaption of the inducer concentration in order to achieve full induction. Still, space time-yield as well as ongoing product formation were favorable for lactose induction, but an optimized fed-batch run was superior in term of productivity to the results derived in chemostats.

**Figure 4:**
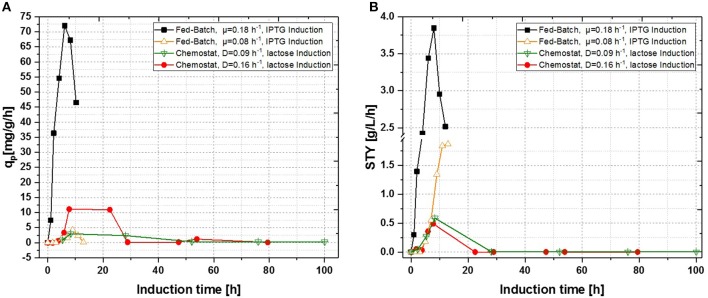
Comparison of a single-stage continuous cultivation of *E. coli* BL21 (DE3) for recombinant inclusion body production (D = 0.16 ± 0.021 h^−1^, pH = 6.7, V = 650 mL, T = 31.5°C, induction with lactose) with fed-batch cultivations (μ = 0.08 ± 0.023 h^−1^ and μ = 0.18 ± 0.01 h^−1^, pH = 6.7, V_end_ = 7.5 L, T = 31.5°C; induction with IPTG). **(A)** Specific productivity as function of induction time; **(B)** space-time-yield as function of induction time.

In a previous study the growth rate seems to correlate with productivity in fed-batch cultivations for the T7-expression system (Hausjell et al., 2018). Previously performed fed-batch cultivations also revealed that short-term productivity can be boosted by an increase of growth rate, leading to optimized induction times of 6–8 h, depending on the growth rate, respectively (Kopp et al., 2018; Slouka et al., 2019). As a growth rate or a dilution rate of D = 0.16 h^−1^ would implement a residence time of 6.25 h^−1^ the average induction time for host cells would be in the same optimum found out in fed-batch cultivations. Supplying more energy by applying higher dilution rates, therefore should boost product formation (Sandén et al., 2003; Boström et al., 2005). Still, higher dilution rates might be critical, as wash out might occur (Paalme et al., 1995). IPTG induction was not tested at higher dilution rates as induction with lactose showed beneficial results in terms of space-time yield. To compensate for the eventually occurring inducer limitation, lactose concentration in the feed was increased according to the previous established results (Figures 4A,B).

**Figure 5:**
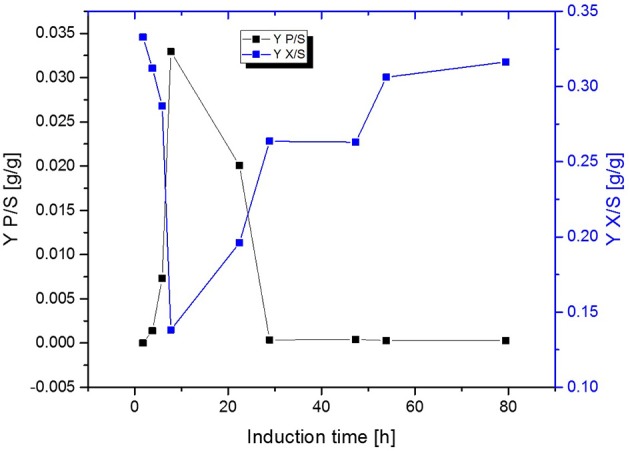
Comparison of the biomass- and product yield throughout induction phase of chemostat operated at D = 0.16 h^−1^ induced with lactose.

Fed-Batch cultivation performed at growth rates of μ = 0.15 h^−1^ during induction phase did also provide far higher production levels compared to a fed-batch operated at μ = 0.1 h^−1^ during induction phase, which is in accordance with literature (Hausjell et al., 2018; Kopp et al., 2018; Slouka et al., 2019) Short-time specific productivity could be also increased in chemostat cultivation, by applying higher dilution rates when compared to previous chemostat cultivations at D = 0.1 [h^−1^], however a constant productivity was not accomplished (compare to Figures 4A,B). Low levels of acetate formation were also detected with at-line measurements (data not shown) once q_p_ decreased, which is in accordance with literature [33]. A comparison of product formation and biomass measurements indicated that simultaneous product formation and biomass production are not capable of being constant over induction time (compare to Figure 5). This was confirmed by the increase in biomass production after 29 h again, hence almost no product formation was detectable at the same time of the process (compare to Figures 4A,B and Figure 5).

Required resources for recombinant protein production such as raw material and energy for the amplification of the foreign DNA and the followed expression of the recombinant protein bear a metabolic burden onto host cells (Hoffmann and Rinas, 2004). As the T7-RNA polymerase system is a very strong expression system, it exerts a high metabolic burden onto the host cells (Heyland et al., 2011). The decrease of DCW during the induction period of the continuous cultivation could therefore be explained due to strong overexpression of the recombinant protein in the beginning of the induction phase, leading to decreased cell growth rates (Scott et al., 2010) and genetic instabilities (Croughan et al., 2015) and consequently to a decrease in productivity.

### Cascaded Continuous Cultivation

All previous results obtained in chemostat cultivations (compare to Figures 3A,B, 4A,B, 5) indicated that biomass production and high protein production cannot be coped within one cultivation step. Therefore, the next approach was to spatially separate biomass production from induction. In order to reduce the metabolic burden, non-induced cell growth and recombinant protein expression were decoupled from each other within this study in subsequent cultivation steps by applying a cascade of two sequentially operated continuous bioreactors as described in Figure 2. A batch phase, similar to single-stage continuous cultivation, in all reactors was followed by a non-induced continuous phase in order to achieve steady state conditions, ensuring the constant transfer of exponentially growing cells from the first reactor to the second before the induction of the recombinant protein expression started. The dilution rate of the first reactor was set to D_1_ = 0.08 h^−1^, while the highly concentrated feed is added at a dilution rate of D_R2_ = 0.02 h^−1^. The flow out of the second reactor therefore was calculated D_2_ = D_1_ + D_R2_, resulting in a final dilution rate for the cascade of 0.1 h^−1^ and 10 h of residence time for cells in the second reactor. As the previous chemostat and fed-batch cultivations showed the trend that a higher growth rate/higher dilution rate correlates to higher product formation this was also considered here. By varying the reaction volume of both reactors implemented in the cascade, the residence time could also be varied, optimizing the generation process for inclusion bodies. Presetting optimal dilution rate was thought to be beneficial for biomass formation in the first reactor and recombinant protein production in the second reactor. The following hypothesis are related to these cultivations:

A higher dilution rate D_2_ implementing a higher growth rate of expressing cells in the second reactor leads to higher space-time yields and specific productivities (Kopp et al., 2017; Hausjell et al., 2018). Variation of the residence time τ, might lead to different effects onto IB-formation as cells might be washed out before metabolic burden decreases levels of specific productivity (Wurm et al., 2017b). Higher inducer concentrations favor higher specific uptake rates and therefore higher product concentrations and space-time yields can be achieved.

#### Biomass of Cascaded Continuous Cultivation

**Figure 6:**
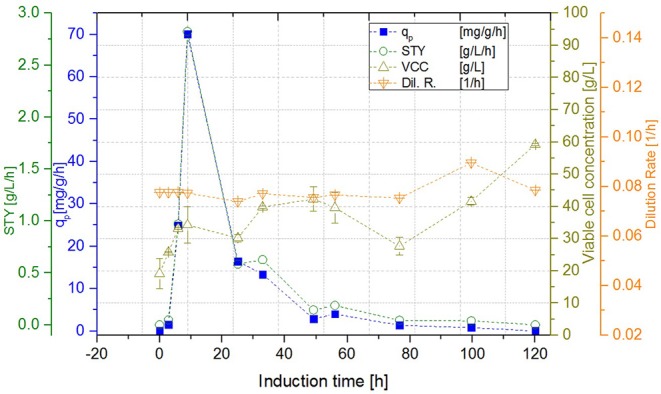
Cascade 2 operated at a dilution rate of D_ges_ = 0.08 ± 0.004 h^−1^ (pH = 6.7, T_R1_ = 35°C, T_R2_ = 31.5°C).

While the DCW of the cascade 1 (induction with IPTG) reached a steady state concentration of 29.99 ± 0.71 g L^−1^, DCW in the second reactor of the cascade 2 (induction with lactose) continued to increase to a maximum of 59.99 ± 0.21 g L^−1^. Cascade 3 showed slightly lower maximum levels of DCW, reaching amounts of 53.31 ± 1.20 g L^−1^. However, DCW measured in the lactose induced cascades (cascade 2, 3) fluctuated during the induction phase, as lactose—unlike IPTG—still was metabolized, which can be seen exemplarily in Figure 6. During these cultivations, the percentage of living cells, determined via flow cytometry (Langemann et al., 2016), was always above 99% (comparison to DCW therefore not shown). Therefore, the decline in productivity is probably not a result of cell death, which was partially monitored in chemostat cultivations, but could have been caused by the high metabolic burden that the product formation implements onto the cells.

#### Productivity of Cascaded Continuous Cultivation

**Figure 7:**
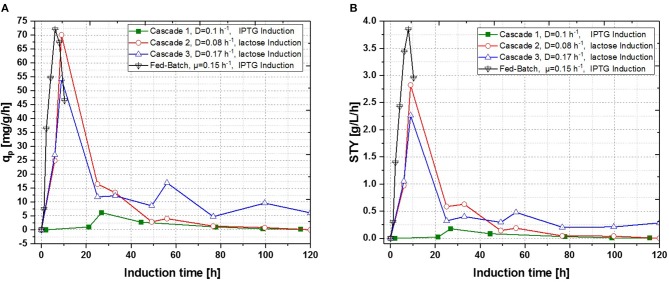
Operated cascades of two reactors compared to an optimized fed-batch for recombinant inclusion body production in *E. coli* BL21 (DE3) with variation of dilution rate and Inducer (pH = 6.7, T_R1_ = 35°C, T_R2_ = 31.5°C). **(A)** Specific productivity as function of induction time; **(B)** STY as function of induction time).

In a recent publication, constant productivity in a cascade cultivation system was observed, when performing induction with IPTG (Schmideder and Weuster-Botz, 2017). In order to test the feasibility of this study to produce an industrial relevant protein, we also performed a cascade cultivation testing IPTG as an inducer. However, results were in accordance with the single-stage cultivations described in the previous section and the specific productivity could not be kept constant over a longer period for IPTG induced cascade cultivations. A maximum q_p_ value of 6.44 mg g^−1^ h^−1^ was reached for the IPTG induced cultivation after 44 h of induction. In case of cascade 1, a later rise of product formation and a later drop of productivity was observed. During induction with IPTG in the cascaded system, the highest STY of was reached after 44 h of induction, shown in Figure 7A. Lactose induction (cascade 2) resulted in a maximum value at the very beginning of the induction phase, comparable to the achieved productivity throughout fed-batches induced with IPTG, reaching q_p_ values of approximately 70 mg g^−1^ h^−1^. Although cascade 2 produced higher amounts than the cascade 1 induced with IPTG, it still follows the same trend, as productivity declines significantly over time and a steady state in terms of productivity was never accomplished. As lactose showed again beneficial results in terms of productivity in cascaded chemostat cultivations, a further alteration of the dilution rate was not considered for IPTG induced cascaded cultivation. During cascade 2 low lactose accumulation was measured with at-line HPLC in the cultivation broth throughout the whole process (data not shown), therefore the lactose concentration was maintained for the mixed feed used for induction of cascade 3. Increasing dilution rate in cascade 3 (induced with lactose) eventually led to a stable productivity after approximately 25 h of induction and showed stable productivity for the ongoing process time. Even though cascade 2, operated at a dilution rate of 0.1 h^−1^, exceeds higher short-term specific productivity, overall productivity and space time yield of cascade 3 are superior to cascade 2.

Results for the different performed chemostat cultivations are presented in Figure 7 (a for specific productivity, b for time space yield) compared to an optimized state-of-the-art fed-batch process. Comparing cascade 1 and cascade 3 with each other after 5 residence times, a significant increase in specific productivity and space-time yield is observed. This might be due to a constant washout of producing cells during continuous operation (Schmideder et al., 2016). The first improved cascaded process could not outperform an optimized fed-batch process (compare to Figure 7 and Table 1) regarding product concentration and product yield coefficient yet. As product formation throughout fed-batches is very much dependent on induction time, high standard deviations can be seen for productivity, as values were calculated as an average over the time of induction. Chemostat processes and cascade 1 and 2 did not show constant productivity over time, explaining high standard deviations seen there. Cascade 3 was the only process to feature constant productivity, therefore lower standard deviations compared to the other processes can be seen. Still we would like to highlight that even though a constant productivity could be reached metabolic fluxes (such as shifts in CDW, dO_2_, and CER shown in Supplementary Figures) occur throughout production. As fed-batch results indicated, that specific productivity declines during an induction time of 12 h, it might be necessary to wash out cells before maximum productivity can be achieved, in order to keep them in a stable production mode. However, when taking sterilization and cleaning times into account, the cascaded process could be a well-used alternative to fed-batch processes in combination with the possibility of creating a fully integrated process.

**Table 1:** Comparison of bioprocesses for inclusion body production with *E. coli* BL21(DE3) in chemostat cultivation, an optimized fed-batch process and continuously operated cascades with different dilution rates and q_s_,_lac_-values regarding cell dry weight (DCW), residence time in the induction reactor (τ), specific glycerol uptake rate (q_s,Glycerol_), product selectivity (Y_P/S_), product yield coefficient (Y_P/X_), space-time yield (STY), and product concentration (C_P_).

**Parameters**	**Fed-batch 1 (μ = 0.08 ± 0.024 h^**−1**^)**	**Fed-batch 2 (μ = 0.18 ± 0.012 h^**−1**^)**	**Chemostat (D = 0.16 ± 0.021 h^**−1**^)**	**Cascade 1 (D_**2**_ = 0.1 ± 0.001 h^**−1**^)**	**Cascade 2 (D_**2**_ = 0.08 ± 0.004 h^**−1**^)**	**Cascade 3 (D_**2**_ = 0.16 ± 0.010 h^**−1**^)**
Induction as IPTG or q_slac_ [g g^−1^ h^−1^]	IPTG, 0.5 mM	IPTG, 0.5 mM	q_slac, max_^*^0.5	IPTG, 0.5 mM	q_slac, max_	q_slac, max_
CDW [g L^−1^]	31.6 ± 4.8	34.9 ± 4.1	13.9 ± 5.4	27.5 ± 4.6	39.6 ± 9.8	37.8 ± 8.9
τ [h]	-	-	5.9 ± 0.2	10 ± 0.1	12.55 ± 0.83	6.0 ± 0.4
q_s,glycerol_ [g g^−1^ h^−1^]	0.19 ± 0.06	0.54 ± 0.1	0.48 ± 0.10	0.22 ± 0.07	0.292 ± 0.05	0.48 ± 0.06
Y_P/S_ [mg g^−1^]	62 ± 78.4	158.9 ± 189.5	9.5 ± 17.4	8.5 ± 9.0	7.3 ± 7.79	12.7 ± 4.3
Y_P/X_ [mg g^−1^]	106 ± 29.3	121.6 ± 67.2	27.2 ± 54.9	22.8 ± 23.2	23.7 ± 23.8	147.2 ± 55.9
STY [mg L^−1^ h^−1^]	825.6 ± 281.1	2411.1 ± 1129.2	89.7 ± 148.6	60.8 ± 64.63	118.5 ± 132.2	311.8 ± 98.6
C_P_ [mg L^−1^]	1448.9 ± 1.065	3969 ± 2854.9	236.07 ± 355.368	736.8 ± 720.3	1607.0 ± 925.3	6139.0 ± 2359.1

*Values are calculated as an average after 5 residence times for continuous applications, while fed-batch-values are calculated at as an average throughout induction phase; cultivations shown in Figures 4A,B are not taken into account of this table, as productivities were very little. Values are calculated as an average after 5 residence times, for the fed-batch they are calculated as an average over induction time. High standard deviation result from time dependent production*.

## Discussion

In order to achieve constant productivity of the IB based product, different cultivation modes were tested. Trying to establish a continuous cultivation mode specific productivity and space time yield of chemostat- and the cascaded cultivation were compared to state-of-the-art fed-batch processes. First optimized process parameters such as dilution rate, residence time and inducer concentration led to the optimized cultivation process seen in Figures 7A,B tested by IB titer during the cultivation. Results indicate, that lactose is the inducer of choice for induction in all continuous operated systems, when compared to IPTG. In previous studies, it has been shown that cultivations of *E. coli* BL21(DE3) carried out on glycerol tend to produce more recombinant protein with a product optimum at a q_s,glycerol_-level seen around 0.3–0.35 g g^−1^h^−1^ (Kopp et al., 2017). Space-time yield and product yield of cascade 3 operated at a dilution rate of D_2_ = 0.16 h^−1^ induced at q_s,lac,max_ showed best results up to date compared to the other continuously operated processes performed within this study. As glycerol needs increased cAMP levels, which are also needed for lactose uptake (Bettenbrock et al., 2006), this might be a key function in regulating higher lactose uptake and subsequently increasing productivity and product titer. Higher dilution rates, such as operated in cascade 3, implemented lower residence times of producing cells, which might help to reduce the metabolic burden onto host cells. Hence high dilution rates will consequently decrease DCW values and ongoing lead to a washout of cells (Paalme et al., 1995; Schmideder et al., 2016). Schmideder and Weuster-Botz (2017) improved the overall process performance producing soluble PAmCherry with *E. coli* in a continuously operated cascade by reducing the overall dilution from 0.2 to 0.13 h^−1^. Dilution rates of 0.2 h^−1^ were not considered within this study but could be investigated in subsequent cultivations in order to further increase the overall process performance. Simulations of plasmid stability in a continuously operated cascade performed by Hortacsu and Ryu (1991) showed that there is an improved plasmid stability at higher dilution rates for long-term cultivations (Park et al., 1990; Hortacsu and Ryu, 1991; Rhee and Schügerl, 1998). Hortacsu and Ryu (1991) also proved that a linear, time-dependent growth rate control, using a slow increase of dilution rate resulted in an increased average productivity when compared to a process operated at a constant growth rate. As a part of this study we show that single vessel chemostat cultivation does not provide stable long-term product formation, when keeping process parameters constant. The cultivation mode proposed by Hortacsu and Ryu (1991), similar to an accelerostat cultivation mode, could therefore be seen as a potential cultivation mode for achieving stable productivity within a single-vessel cultivation mode (Paalme et al., 1995). We were able to show, that cascade system implemented by Schmideder and Weuster-Botz (2017), is very well-suitable to increase continuous microbial processes regarding the space-time-yield. Spatial separation from biomass growth and recombinant protein production clearly seems to be beneficial for process performance. Application of higher dilution rates throughout cascade processes resulted in higher product titers, we hypothesize that shorter residence times in the producing reactors seems to be highly favorable for recombinant protein production. On the other hand, too high dilution rates (lager than 0.25 h^−1^) might lead to a “washout-effect” of the host cells, so a tradeoff must be found between optimal feeding and optimal residence time. As highest productivity of a fed-batch is generally found after 6–8 h, residence times in this range may be optimal for cascaded cultivations. Therefore, using higher dilution rates smaller than 6 h may result in lower productivity as not enough time for induction is given in the reactor. As biological evolution throughout continuous cultivations might always lead to certain gene up- or downregulations (Wick et al., 2001; Peebo et al., 2015) the results indicate that cells tend to stay in a rather stable system when they are exhibiting lower residence times in a cascaded system. Taking the different amounts of recombinant protein produced as an indicator for changes in metabolome or transcriptome, the cascaded system seems to keep cells in rather stable conditions, when compared to common chemostat cultivation.

During a previous work conducted in fed-batch experiments, we were able to show that cell lysis is occurring at elongated induction times, when induced with IPTG (Kopp et al., 2018; Slouka et al., 2019). As lactose is a rather “soft” inducer and is used in combination with a relatively short average residence time of 6.25 h throughout cascade 3, this is possibly the quintessence for stable protein production within this process. As a continuous process for recombinant protein production can either be improved by increased titer concentrations in the bleed or by increasing $\frac{{dV}_{Bleed}}{dt}$, cascade 3 operated at q_s,lac,max_ with D_2_ = 0.16 h^−1^ combines both features of a possible improvement and could therefore be a well-suited cultivation system, when implementing a fully integrated continuous process.

In this work, the economical and practical feasibility of a continuous cultivation process to produce a recombinant protein as IB with the genetically modified strain *E. coli* BL21(DE3) was investigated. The overall goal was to provide a promising alternative to optimized fed-batch processes, trying to eliminate time-dependent changes of productivity. It was shown that a spatial separation of biomass formation and recombinant protein expression by setting up a cascade of two stirred tank reactors significantly improves the overall process performance of a continuous cultivation. It was further confirmed that the application of lactose as an inducer instead of IPTG resulted in higher productivities over time in chemostat and in cascaded cultivation. Usage of lactose as an inducer instead of IPTG in combination with a cascaded cultivation mode at optimized dilution rates leads to an increase in specific productivity and space-time yield of the cascaded system when compared to chemostat processing. As higher dilution rates also seem to boost titer formation, the overall mass flow of product out of the cascade was significantly increased. Furthermore, continuous processing is referred to boost the time-space-yield, when taking set-up- and cleaning times into account. As a continuous cultivation mode also provides the possibility of achieving time-independent critical quality attributes, downstream processing should be eased and more robust, consequently. Therefore, we want to highlight, that the optimized cascaded run could potentially fulfill the criteria of an industrial continuous cultivation system. We are currently optimizing the given system in order to achieve a stable long-lasting process, which can outperform the fed-batch as golden standard.

## Data Availability Statement

The datasets generated for this study are available on request to the corresponding author.

## Author Contributions

JK, A-MK, and PV performed the cultivations. CS and CH supervised the work and gave the valuable input. JK, CS, OS, and CH drafted the manuscript.

### Conflict of Interest

The authors declare that the research was conducted in the absence of any commercial or financial relationships that could be construed as a potential conflict of interest.
