# Supplementary material for: Boosting Recombinant Inclusion Body Production—From Classical Fed-Batch Approach to Continuous Cultivation
Source: Front Bioeng Biotechnol. 2019 Oct 31;7:297. doi: 10.3389/fbioe.2019.00297 (PMC6834550; doi:10.3389/fbioe.2019.00297)
Supplement: Supplementary file 1 [file Data_Sheet_1.docx]

# Supplementary Figures:


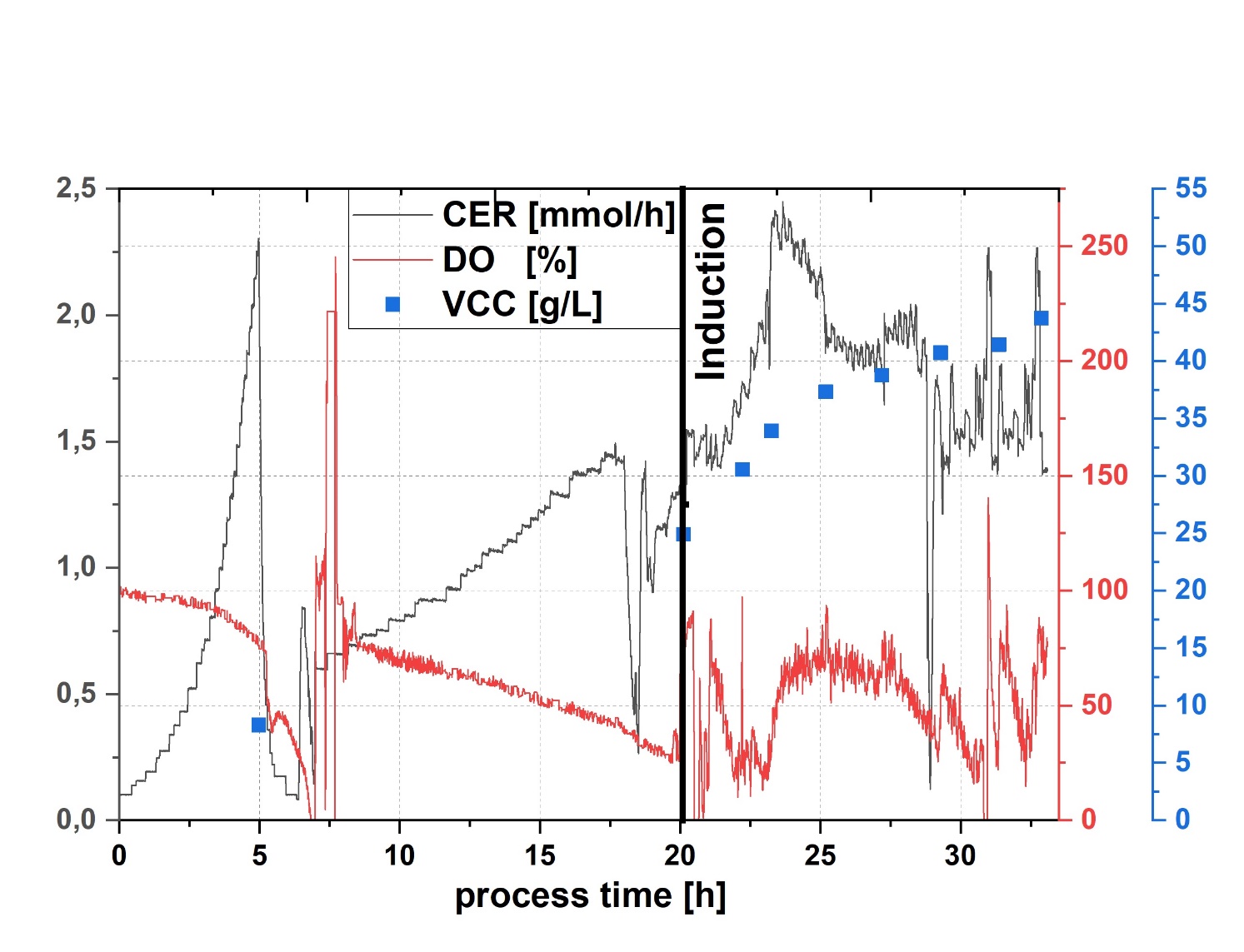


**S1:** Process data of a performed fed-batch process for the given recombinant protein. Induction lasted for 12 h with a µ of 0.18 h^-1^. VCC is the viable cell concentration calculated via flow cytometry analysis. CER is the carbon evolution rate, DO the dissolved oxygen.


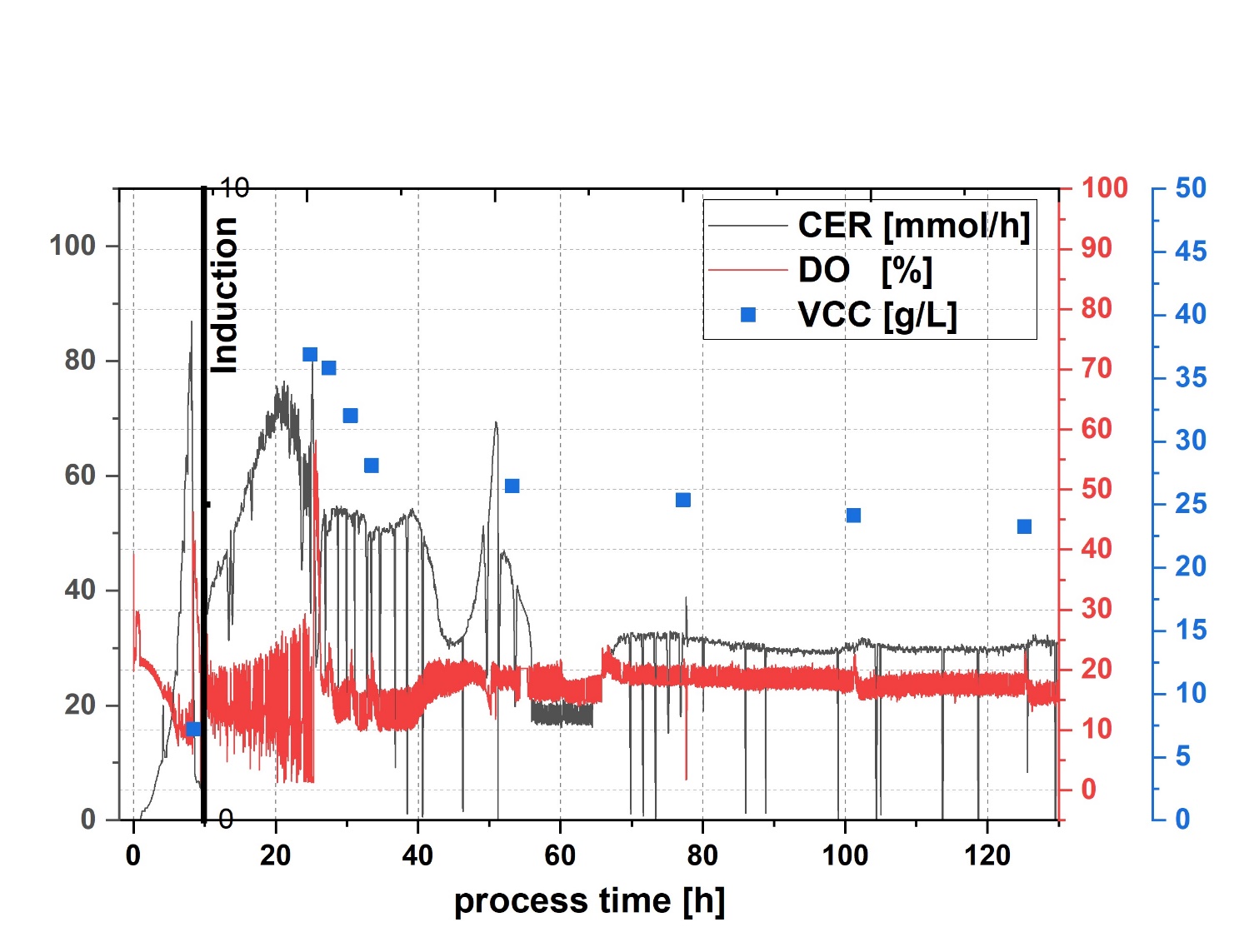


**S2:** Process data of a performed one-pot chemostat process for the given recombinant protein. Dilution rate was 0.09 h^-1^. Induction was performed with lactose.


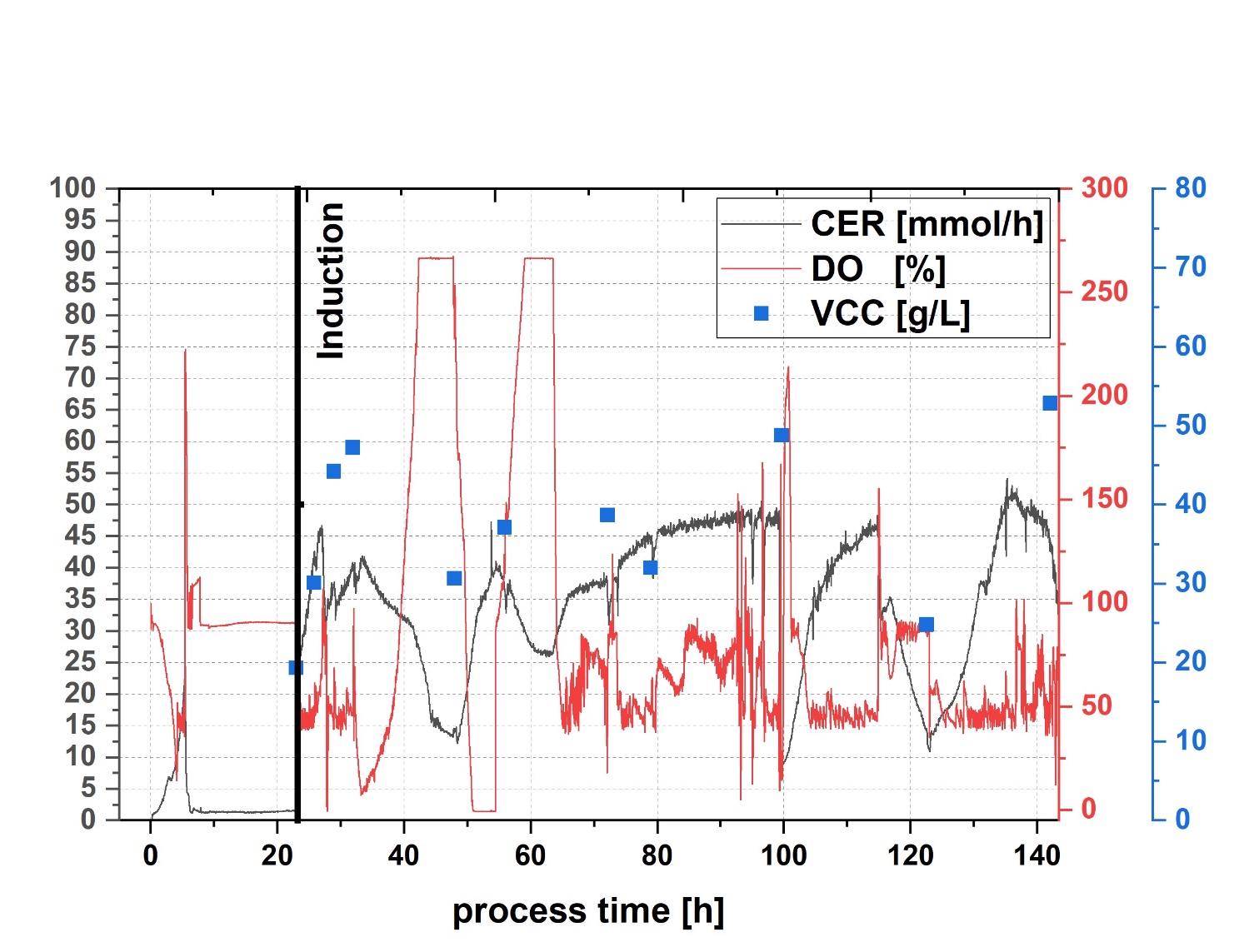


**S3:** Process data of a performed cascaded chemostat process for the given recombinant protein. Dilution rate was 0.16 h^-1^. Induction was performed with lactose.
